# The Role of the Equestrian Professional in Bridle and Bit Fit in the United Kingdom

**DOI:** 10.3390/ani14223188

**Published:** 2024-11-06

**Authors:** Kathryn Nankervis, Jane M. Williams, Diana Fisher, Russell MacKechnie-Guire

**Affiliations:** 1 Equine Department, Hartpury University, Gloucester GL19 3BE, UK; jane.williams@hartpury.ac.uk; 2 Woolcroft Equine Services, May Lane, Wisbech PE13 5BU, UK; ceo@mastersaddlers.co.uk

**Keywords:** bridle, bit, fitter, fit, equine musculoskeletal therapist, equestrian coach, qualification, equine welfare

## Abstract

To ensure the comfort of ridden horses, equipment including bridles and bits should be suitable and fit correctly. Horse owners employ a variety of equestrian professionals with varying degrees of responsibility regarding bridle and bit fit. This study aimed to compare the frequency and manner in which equestrian professionals within a multidisciplinary team (MDT) engage with clients on matters of bridle and bit fit, and to capture their perceptions of the key issues in bridle and bit fit. The participants completed an online questionnaire to obtain information regarding participant demographics, bridle fit and bit fit for the horse. Descriptive statistics and Kruskal–Wallis analyses were performed (*p* ≤ 0.05). Inductive content analysis identified themes from open-question responses. The results from 377 respondents were analyzed: 184 saddle fitters, 116 coaches and 77 therapists. The results showed that 65% of coaches ‘nearly always’ or ‘very often’ ask clients when they last had their bridle fitted, which was significantly more than saddle fitters (*p* = 0.002). More saddle fitters assess bridle fit statically and dynamically compared with coaches (*p* = 0.02). ‘Browband too small’ featured in the top three bridle fit issues for all professions. More professionals reported issues of bit suitability than fit. The findings demonstrate that whilst various equestrian professionals assess and act upon issues of bridle and bit fit, the extent to which this occurs is lower than for the saddle. Further work should quantify the prevalence of issues in bridle and bit fit and suitability in ridden horses.

## Simple Summary

Horse owners employ a variety of equestrian professionals, e.g., coaches, therapists and fitters, all of whom play a role in safeguarding equine welfare. Bridle and bit suitability and fit contribute to the horse’s comfort and performance, and, hence, equestrian professionals forming a multidisciplinary team (MDT) have opportunities to support the horse owner on matters of bridle and bit fit. The aim of this study was to learn more about the frequency and nature of observations of bridle and bit fit made by professionals and the type of issues they see most often. The participants completed an online questionnaire designed to obtain information about their professional role and experience, whether they carried out assessment of bridles and bits as part of their professional role and what they perceived as the key issues of fit. Data were analyzed descriptively and statistically (*p* ≤ 0.05). The results from 377 respondents were analyzed: 184 saddle fitters, 116 coaches and 77 therapists. Of the three professions, coaches were found to assess their clients’ bridle and bit fit more than saddle fitters and therapists. Coaches and therapists agreed on the top three bridle fit issues, which were ‘browband too small’, ‘noseband too tight’ and ‘noseband too low’. The professionals reported that bits may not fit or be suitable for the horse and rider combination. Saddle fitters, coaches and therapists can all support horse owners in achieving optimal bridle and bit fit.

## 1. Introduction

In most equestrian activities, a bridle is used to provide an interface between horse and rider for communication of the rider’s signals to the horse. Some form of restraint being applied to the head of the horse has been evident from early records of its domestication [1]. Via the bridle, forces are transmitted from the riders’ hands through the reins to both sides of the mouthpiece of the bit. The bit design, function and materials vary, but it generally takes the form of a cylindrical bar, either jointed or unjointed and positioned within the inter-dental space. The commonly used snaffle bridle usually comprises a headpiece, browband, cheekpieces, throat lash, noseband, bit, and reins. In contrast to a large body of evidence relating aspects of saddle fit and saddle pressures to the movement of the horse [2,3,4,5,6,7,8,9,10], and several reviews which highlight the importance of saddle fit for the avoidance of injury and poor performance [11,12,13,14,15], there is rather less evidence relating aspects of bridle fit and/or pressures to either biomechanics, injury risk or performance [16,17,18,19]. In the interest of equine welfare, however, significant attention has focused on the use of nosebands which do not allow for jaw movement during exercise [20,21,22], and rider perceptions and behavior regarding noseband use [23,24,25]. To date, research has been carried out on the action of bit under experimental conditions [26,27,28] and the impact of bit selection, fit and/or use in various types of competition horses [29,30]. Given the proximity of the bridle to prominent bony and sensitive structures of the head such as the caudal aspect of the ear, the wing of the atlas, the temporomandibular joint, trigeminal nerve and the position of the bit within the mouth [31], bridle and bit design, suitability and fit warrant equal scrutiny as saddle design, suitability and fit in both research and practice.

Horse owners often enlist the services of a qualified saddle fitter to fit their saddle [32] but appear less likely to engage a qualified bridle fitter regarding certain aspects of bridle selection and fit. A recent study found that 71% of riders responding to an online survey would consult their trainer, coach or online sources such as YouTube for guidance on noseband fit [25], with fewer than 25% reporting that they would consult a bridle fitter. Bridle- and bit-fitting qualifications have been developed more recently than saddle-fitting qualifications in the UK. At the time of writing, there were 34 bridle fitters registered with the Society of Master Saddlers and 315 qualified saddle fitters [33]. The vast range of bridles and bits available present a complex landscape for the horse owner to navigate, creating the need for support from experienced, knowledgeable individuals to safeguard equine welfare and rider safety including individuals specifically trained in bridle and bit fit.

Within human healthcare, a multidisciplinary team (MDT) is defined as a team of healthcare professionals working to improve care and health outcomes [34]. Successful MDT approaches are evident in equine healthcare [35,36,37] supporting a rationale for engaging the whole MDT in raising standards in saddlery fit, where saddlery includes saddle, bridle and bit. Recently, an online survey on the role of the equestrian professional in saddle fit found that 61% of coaches and 100% of equine musculoskeletal therapists “nearly always” or “very often” ask clients when they last had their saddle fitted [32]. In total, 61% of coaches and 65% of therapists reported that they would make an assessment of saddle fit as part of their professional role [32]. Despite this apparent vigilance with regard to saddle fit, evidence suggests that saddle fit is often suboptimal [38,39]. Seasonal and developmental changes in the musculature of the horse’s back over the course of a year can impact saddle fit, and so saddle fit should be checked several times a year [40]. Bridle and bit fit is not subject to such fluctuation over time, and so if optimal fit can be achieved for all bridle/bit combinations worn by a horse, it is likely to have a sustained impact on the comfort and performance of the individual. Despite the lack of a specific qualification in bridle and bit fitting, various members of the MDT will encounter bridle and bit fit issues as a result of observations made as part of their normal professional role, as appears to be the case with saddle fit [32]. This overlap in professional responsibility for bridle and bit fit could be a highly effective safeguard to the achievement of optimal bridle and bit fit for the horse. Improving our knowledge regarding the key issues in bridle and bit fit as perceived by various MDT members is a useful practical step towards improving the effectiveness of the MDT in raising the level of bridle and bit fit.

The objectives of this study were to understand how various equestrian professionals assess bridle and bit fit, what bridle and bit fit issues they see most frequently and how often they make alterations to their clients’ bridle or bit to improve fit. It was hypothesized that there would be commonality between professionals in terms of the most frequent bridle and bit issues seen.

## 2. Materials and Methods

### 2.1. Participants

The participants (*n* = 483) were recruited online by sharing the survey link with UK industry regulatory bodies (Association of Chartered Physiotherapists in Animal Therapy, Register of Animal Musculoskeletal Therapists, British Horse Society, Society of Master Saddlers and British Equine Veterinary Association), national press (Horse and Hound, published online, April 2022) and social media (Facebook^®^, Twitter^®^, Linked In^®^, all CA, USA) groups and pages. The inclusion criteria required participants to be over 18 years of age and be practicing in one of the equestrian professions targeted, i.e., veterinarians, physiotherapists, chiropractors, massage therapists, coaches, farriers, dentists, and saddle fitters.

### 2.2. Survey Design

This study was designed as an online survey (JISC online surveys, Bristol, UK) with 25 closed questions, which included 11 closed questions and 14 multiple-choice questions containing Likert Scale, 6 ranking and 9 open text questions (Supplementary File S1). The survey was split into three major sections: (1) participant demographics, (2) saddle, bridle and bit fit for the horse, and (3) saddle fit for the rider. The results from Sections 2 and 3 relating to questions on saddle fit for the horse and rider were presented elsewhere [32]; so, the results presented here relate to Section 1 and the questions in Section 2 relating to the bridle and bit. Section 1 questions explored the professional’s main profession, country of residence, their professional memberships/affiliation and if qualified, how long they had been qualified? In Section 2, the professionals were asked how frequently they asked clients when they last had their bridle and bit fitted and whether they check the qualifications of the person who fitted the bridle and bit, whether they themselves assess the bridle and bit fit as part of their professional service and the manner in which they carry out their assessment. They were also asked about the most frequent bridle- and bit-fitting issues they encounter, and whether they make alterations to the client’s existing set-up to improve bridle and bit fit.

The draft survey was pilot-tested by 13 equestrian professionals for useability and edited to correct any errors before being launched. The survey was live for 68 days and 80% of the responses were obtained within the first fourteen days. A priori sample size calculation (Survey Monkey™, Seattle, WA, USA), identified from a population estimate of 18k [41] that a minimum of 377 responses were required to be representative of the targeted professional populations at the 95% confidence level, with a ±5% margin of error.

#### 2.2.1. Descriptive Analysis

Data were exported from JISC to Microsoft Excel™ Version 2020 (Redmond, WA, USA). Frequency analysis identified respondent role, if they held qualifications, and how long they had been qualified in their respective professions, as well as how many horses they visited weekly. Additional frequency analyses reported how different professions approached client visits with respect to assessing bridle and bit fit, how they guided clients for future visits and the most frequently encountered issues rated as follows; 1: nearly always, 2: very often, 3: often, 4: not often, 5: never. “Nearly always” was defined as >90% of the time, “very often” as 60–89% of the time, “often” as 30–59% of the time and “not often” as 1–29% of the time. Data met non-parametric assumptions; therefore, a series of Kruskal-Wallis analyses were used to identify whether differences occurred across the cohort, i.e., between the respondent professions, and whether differences existed between specific groups, i.e., saddle fitters versus equestrian coaches versus therapists in terms of the number of horses seen per week, number of years qualified, their approach to bridle and bit fit assessment and the most frequent bridle and bit issues encountered. Therapists included all musculoskeletal therapists, i.e., veterinary physiotherapists, osteopaths, chiropractors, and massage therapists.

For factors where significant differences were found, Mann-Whitney U post hoc tests identified how ratings differed between the groups. Median rankings for individual factors were examined to identify the direction of differences between disciplines; where median values were the same, mean rank differences obtained from post hoc tests differentiated between disciplines. Statistical analyses were carried out in SPSS version 27 and significance was set at *p* < 0.05.

#### 2.2.2. Inductive Content Analysis

For open text responses, inductive content analysis, using an open coding approach, was applied to create emergent categories that described the participants’ perspectives and increased knowledge and understanding related to the process of bridle and bit fit.

## 3. Results

### 3.1. Demographics

A total of 483 respondents completed the survey, with responses coming from Europe, Central/South America, and North America. The majority of respondents (*n* = 377) who completed the survey were based in the UK (6% margin of error at 95% CI) and comprised saddle fitters, coaches and therapists. No responses were obtained from veterinarians, equine dentists or farriers. Factors influencing professional practice can vary by country and culture; therefore, to ensure data represented a homogenous population, UK-only responses were analyzed and are reported forthwith. The majority of respondents (92.3%; *n* = 276) held qualifications for their profession; 57.9% (*n* = 172) of respondents had been qualified for more than 11 years. Most (69.0%; *n* = 203) of the sample saw less than 20 horses per week. The client base of the respondents took part in a wide range of equestrian disciplines.

A total of 184 saddle fitters completed the survey (4% margin of error at 95% CI); 87% (*n* = 160) were qualified, with the remaining 13% (*n* = 24) not qualified. Saddle fitters had typically (i.e., the most frequent response) been qualified between 11 and 20 years (28% and *n* = 52) and most frequently visited between 11 and 20 horses per week (47% and *n* = 87). A total of 116 coaches completed the survey (9% margin of error at 95% CI). Ninety-four per cent (*n* = 109) held a professional qualification, and 87% (*n* = 101) had been qualified for >5 years. Coaches typically saw between 11 and 20 horses per week (61%; *n* = 71). A total of 77 therapists completed the survey (11% margin of error at 95% CI); all (100%) held professional qualifications. Fifty-six per cent (*n* = 43) of therapists had been qualified for >5 years. Therapists typically saw between 11 and 20 horses per week (64%; *n* = 49). No significant difference was found in qualified status between saddle fitters, coaches and therapists (*p* = 0.103). However, there were significant differences in the number of years fitters, coaches and therapists had been qualified (*p* = 0.004). Post hoc tests showed fitters (80% >5 years) had been qualified longer than therapists (*p* = 0.007; 56% >5 years) and therapists had been qualified for less time than coaches (*p* = 0.0004; 87% >5 years). Cumulatively, significant differences existed between the number of horses seen per week between saddle fitters, therapists and coaches (*p* = 0.002). Post hoc tests identified that saddle fitters saw more horses per week (73% visit > 10 horses/week) than therapists (*p* = 0.042; 64% visit > 10 horses/week) and therapists saw more horses per week than coaches (*p* = 0.001; 61% visit > 10 horses/week).

### 3.2. Bridle and Bit Assessment

In total, 39% of saddle fitters (*n* = 72), 64% of coaches (*n* = 74) and 25% of therapists (*n* = 19) “nearly always” or “very often” asked clients when they last had their horses’ bridle fitted. Post hoc tests found coaches (47%; *n* = 55) “nearly always” asked clients when they last had their bridle fitted, which was more frequently than saddle fitters (*p* = 0.002; 20%; *n* = 37). A total of 25% of saddle fitters (*n* = 46), 65% of coaches (*n* = 75) and 23% of therapists (*n* = 18) “nearly always” or “very often” asked clients when they last had their horses’ bit fitted but no significant differences were found between groups (*p* = 0.862).

In total, 19% (*n* = 35) of saddle fitters, 30% (*n* = 35) of coaches and 36% (*n* = 28) of therapists “nearly always” or “very often” asked clients about the qualifications of the person who fitted their horses’ bridle. A total of 17% (*n* = 32) of saddle fitters, 34% (*n* = 40) of coaches and 33% (*n* = 25) of therapists “nearly always” or “very often” asked clients about the qualifications of the person who fitted their horses’ bit. No significant differences were found between professions in the frequency with which they asked their clients about the qualifications of the person who fitted their bridle (*p* = 0.397) or bit (*p* = 0.083).

In total, 52% (*n* = 96) of saddle fitters, 64% (*n* = 74) of coaches and 25% (*n* = 19) of therapists “nearly always” or “very often” assess bridle fit as part of their professional service. Fewer therapists assessed bridle fit than saddle fitters (*p* = 0.0004) and coaches (*p* = 0.0004). Furthermore, 38% (*n* = 70) of saddle fitters, 65% (*n* = 75) of coaches and 20% (*n* = 15) of therapists “nearly always” or “very often” assessed bit fit, with coaches assessing bit fit more than saddle fitters and therapists (*p* = 0.0004 for both) and saddle fitters assessing bit fit more than therapists (*p* = 0.021).

Forty-four percent of saddle fitters (*n* = 81) assess bridle fit when the horse is “in the stable and when ridden”. In addition, 47% (*n* = 55) of coaches assess bridle fit “in the stable”, and 57% (*n* = 44) of therapists assess the fit of the bridle “depending on the circumstances of the assessment”. Saddle fitters were found to assess bridle fit in the stable and when ridden with a greater frequency than coaches (*p* = 0.02). Therapists were also significantly more likely than saddle fitters (*p* = 0.008) and coaches (*p* = 0.0004) to assess bridle fit based on the circumstances of the assessment. Significant differences also existed between saddle fitters, coaches and therapists in terms of how they assessed bit fit. Most often, coaches (47%; *n* = 55) assessed bit fit in the stable, whereas for saddle fitters and therapists (40%, *n* = 74 and 52%, *n* = 40, respectively), the manner of their assessment was most often based on the circumstances of the assessment (both *p* = 0.0004).

When recommending a bridle or bit fitter, 89% of saddle fitters (*n* = 164) would normally recommend that their clients consult a specific individual if issues arise with their bridle. A total of 69% (*n* = 53) of therapists and 45% (*n* = 52) of coaches would recommend that their clients consult a qualified professional if issues arise with their bridle. Ninety percent of saddle fitters (*n* = 166) would normally recommend that their clients consult a specific individual if issues arise with their bit. In addition, 67% (*n* = 52) of therapists and 45% (*n* = 52) of coaches would recommend that their clients consult a qualified professional if issues arise with their bit. No significant differences were found in the approach taken by saddle fitters, therapists or coaches when advising clients on bridle and bit fit (*p* > 0.05), whether respondents recommend the clients’ existing bridle or bit fitter (*p* > 0.05), for recommending a specific bridle fitter (*p* > 0.05) or for not making a specific recommendation (*p* > 0.05). However, differences were found between saddle fitters, coaches and therapists when recommending clients to use a specific bit fitter (*p* = 0.017). Saddle fitters (90%, *n* = 166) were found to recommend a specific bit fitter more than coaches (23%, *n* = 27; *p* = 0.0004).

### 3.3. Bridle and Bit Alterations

Sixty-four percent of saddle fitters would “sometimes” alter the noseband (*n* = 118), browband (62%; *n* = 113), cheekpiece (59%; *n* = 108), or headpiece (54%; *n* = 99) to improve bridle fit. Seventy-eight percent of coaches would “sometimes” alter the noseband (*n* = 90), cheek piece (61%; *n* = 71), and parts of the noseband (53%; *n* = 61), browband (47%; *n* = 54) and headpiece (30%; *n* = 35). Twenty-nine percent of therapists (*n* = 22) would “sometimes” alter the bridle by removing the noseband, and browband (20%; *n* = 15) and headpiece (19%; *n* = 15). Significant differences were found in the frequency of alterations made by saddle fitters, coaches and therapists to their clients’ existing bridle setup; specifically, this included alterations made to the headpiece, browband, noseband, cheek pieces, noseband pieces or reins (*p* < 0.0004). Post hoc analysis showed therapists would alter the headpiece and browband less than saddle fitters and coaches (all *p* = 0.0004), and coaches were found to alter the browband more than therapists (*p* = 0.0004). Saddle fitters altered the noseband more than therapists and coaches (*p* = 0.0004 and *p* = 0.003, respectively), and therapists altered nosebands less than coaches (*p* = 0.0004). A similar pattern was observed for altering cheek pieces, with saddle fitters altering these more than therapists (*p* = 0.0004), and therapists altering them less than coaches (*p* = 0.0004). When altering noseband pieces, saddle fitters altered these more than therapists (*p* = 0.0004) and coaches (*p* = 0.027), and therapists altered them less than coaches (*p* = 0.0004). Finally, saddle fitters altered the reins more than therapists and coaches (*p* = 0.0004 and *p* = 0.01, respectively), and therapists altered reins less than coaches (*p* = 0.0004). When altering the bit, saddle fitters, coaches and therapists had significantly different approaches (*p* = 0.0004). Post hoc analyses found saddle fitters made alterations to bit fit more than therapists (*p* = 0.002) and coaches (*p* = 0.0004), and therapists made alterations to bit fit less than coaches (*p* = 0.0004).

### 3.4. Bridle- and Bit-Fitting Issues

**Figure 1:**
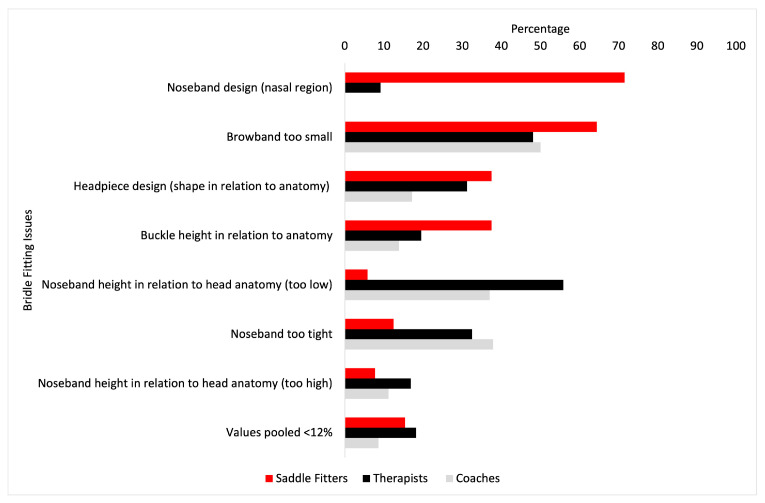
The most frequent bridle fit issues encountered (%) by saddle fitters (red bars), therapists (black bars) and coaches (grey bars). Values pooled <12% were browband too big, browband attachment to the headpiece in relation to anatomy, headpiece design (material), noseband design, noseband width, and noseband padding (not enough/too much).

**Figure 2:**
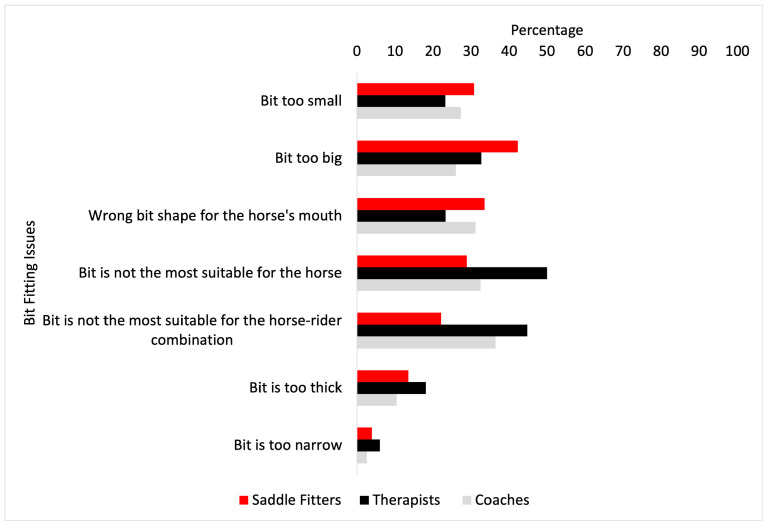
The most frequent bit fit issues encountered (%) by saddle fitters (red bars), therapists (black bars) and coaches (grey bars).

The most frequent bridle fit issues encountered can be seen in Figure 1. There were no significant differences between saddle fitters, coaches and therapists in the most frequent bridle fit issues encountered (*p* > 0.05). No significant differences were found between saddle fitters, therapists and coaches for the most frequent bit-fit-related issues (*p* > 0.05) (see Figure 2), which were the bit being “too big”, “not suitable for the horse” and “not suitable for the horse- rider combination”, with the exception of finding the bit “too small” (*p* = 0.034), where saddle fitters experienced the bit being too small more than coaches (*p* = 0.0004), but no other differences were found between saddle fitters and therapists, or therapists and coaches (*p* > 0.05).

### 3.5. Open Text Questions

**Figure 3:**
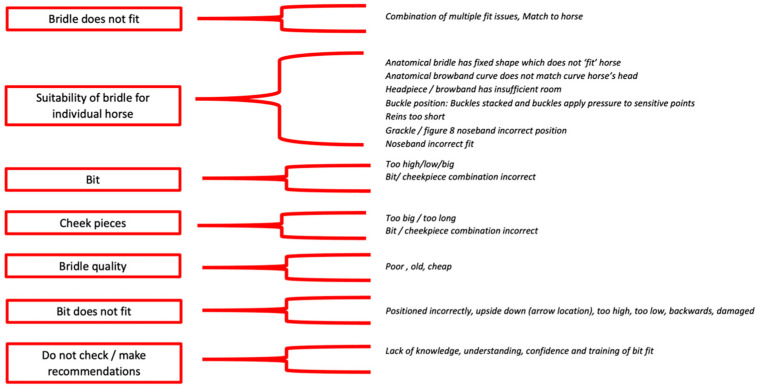
The seven higher-order themes (red boxes) and a summary of the respondents’ text descriptions in relation to bridle and bit issues.

The respondents identified seven themes relating to bridle- and bit-fitting issues encountered: (1) bridle does not fit; (2) suitability of bridle for individual horse; (3) bit; (4) cheek pieces; (5) bridle quality; (6) bit does not fit; and (7) do not check/make recommendations (Figure 3).

## 4. Discussion

The objective of this study was to understand how professionals within an MDT interact with their clients and other professionals on matters of bridle and bit fit and to understand what they perceive to be the key issues in bridle and bit fit. The results give an insight into the current practice of saddle fitters, therapists and coaches with regard to bridle and bit fit in the UK. The hypothesis regarding commonality amongst professions in key bridle and bit fit issues despite differences in the nature of assessment is accepted for the bridle since there were no significant differences between saddle fitters, therapists and coaches in the most frequent bridle fit issues. It is not accepted as regards to the bit since saddle fitters found the bit to be too small significantly more often than coaches.

Previously, we have shown that saddle fitters and therapists were more likely to ask their clients when they last had their saddle fitted than coaches [32]. This is reversed in the current study, with coaches “nearly always” asking their clients when they last had their bridle fitted, more so than saddle fitters and therapists. Coaches are primarily concerned with the correct training of the horse, which is indicated in part by the head ‘remaining in a steady position’ during ridden work [42]. Unsteady head carriage may indicate the horse is either not fully accepting, or able to interpret, the rider’s aids [43]; experiencing discomfort in relation to the equipment used; ill health or some combination of these factors. For example, a bit which is well fitted may cause discomfort during ridden work if the horse has a primary dental issue [44]. The head carried to one side or repeated changes in head position may indicate musculoskeletal pain or other physical discomfort [45]. Whilst the coach is not trained or qualified to determine if problems in head carriage are due to pain, they may feel able to eliminate a training or overt equipment issue in order to expediate referral to a veterinary surgeon or qualified fitter if necessary. Saddle fitters were less likely than coaches to enquire about whether their clients had had their bridle fitted, perhaps reflecting greater interest of the coach in bridle fit from a performance and safety perspective.

As part of the British Horse Society training pathway for coaches, candidates are taught to assess the tack and equipment primarily for safety, but also to check the fit. The results reflected this, with most coaches assessing the fit of the bridle and bit as part of their professional service and reporting that these assessments took place in the stable. Thus, the coach’s assessment of the bridle and bit is generally not carried out during ridden work, or at least their perception of ‘assessment of fit’ does not require the horse to be ridden, demonstrating that coaches take a similar approach to assessment of bridle and bit as they do to assessment of the saddle [32]. Previously, we have shown that 75% of therapists will make an assessment of the saddle [32]; however, in the current study, only 25% of therapists “nearly always” or “very often” assess the fit of the bridle and bit and do so in a manner which “depends on the circumstances of their visit”. Since the therapist’s role is to assess the status of the horse’s musculoskeletal system in horses often undergoing maintenance treatment and/or rehabilitation, ridden observations are perhaps not always warranted or possible. When ridden assessment is possible, assessment of the horse’s behaviors when being tacked up [46] and ridden [45] and comparison of the two may be highly relevant to the therapist’s clinical reasoning. Saddle fitters assess the bridle and bit more frequently than therapists, and when doing so, saddle fitters assess the bridle in the stable and when ridden. This suggests that saddle fitters follow a similar static and dynamic approach to fitting a saddle [32]. Interestingly, unlike bridle and saddle fit, when assessing the fit of the bit, saddle fitters are influenced by the circumstances of their assessment, which may imply that the same level of importance is not afforded to the static and dynamic fit of the bit, or that the saddle fitters are respectful of their professional boundary as bit fit is not specifically part of the saddle-fitting qualification.

Previously, we have shown that 49% of saddle fitters, 77% of therapists and 55% of coaches participating in our survey ‘nearly always’ or ‘very often’ ask about the qualifications of the person who fitted the saddle [32]. The results of this study show that approximately half the number of professionals enquire about the qualifications of the person who fitted the bridle or bit. Qualifications in bridle and bit fitting are relatively new, with most only becoming available within the last 5–10 years; so, this current practice is not surprising. However, when making recommendations, therapists (69%) and coaches (44.8%) recommend that their clients consult a qualified person, suggesting that credence is given to industry-recognized qualifications; however, as of yet, equestrian professionals, and presumably horse owners are less aware of them. Since the introduction of saddle-fitting qualifications in the UK in the 1990s, saddle fitting has evolved considerably, with correct saddle fit widely acknowledged as a crucial part of horse husbandry. ‘Tack and Equipment’, which includes the bridle and bit, is cited by the FEI Ethics and Wellbeing Commission Report 2023 [47,48] as having direct welfare consequences. Similar to the introduction of saddle-fitting qualifications, the introduction of bridle- and bit-fitting qualifications and voluntary regulation of ‘fitting’ via the Equine Fitters Council [49] should result in improvements in standards at all levels of equestrian activity.

Coaches and therapists agreed on the top three issues regarding bridle fit, i.e., “noseband too tight” and “noseband too low”, albeit with differing frequencies of occurrence between professions. Saddle fitters also reported “browband too small” as a key issue but selected other answers, i.e., ‘design’ of nosebands and headpieces, as key issues. Interestingly, when looking at the top three bridle-fitting issues encountered by the respondents, the noseband in some aspect was identified five out of nine times. Whilst ‘browband too small’ may be a direct reflection of the use of an inappropriate size browband, it may also be secondary to suboptimal design of other elements of the bridle. Nevertheless, despite differences in the process of assessment, the commonality of cross-disciplinary observations is encouraging for seeking improvement in fit. If more than one member of the MDT is able to identify and highlight issues in fit to horse owners, there is scope to use the collective MDT to raise standards in bridle fit.

On issues of bit fit, coaches and therapists reported issues regarding ‘suitability’ more often than issues of fit per se. Further work could involve quantitative observations of bridle and bit fit within a general ridden horse population to ascertain how much bridle and bit fit deviates from what is considered optimal. The survey found that saddle fitters and coaches were more likely than therapists to intervene in making alterations to the noseband, cheekpieces and reins ‘if required’ with the intention of improving the fit. Many of the issues identified within the open text responses reinforced earlier question responses, with the addition of generic statements of ‘bridle does not fit’ and ‘bit does not fit’, raising an interesting question about bridle and bit sizing, and whether standard commercially available bridles and bits successfully accommodate the majority of horses. The responses also suggested that since components of the bridle and bit in combination can lead to inappropriate fit, neither the bridle nor the bit can be considered in isolation.

This study had limitations in that some of the questions appeared repetitive for certain professions; for example, if a respondent answered ‘never’ to the primary question “do you make an assessment of bridle or bit fit as part of your professional role” they had to continue to answer in the negative to related questions until they reached the next relevant question. This was deemed preferable to generating separate streams within the survey and reducing the number of responses to each question. It was not always possible to differentiate between components of the question where questions asked, for example, if the professionals “added, altered or removed” items of the bridle. Some of the multiple-choice and open text questions produced responses which overlapped, and like all survey-based studies, the respondents were motivated to participate and may not represent the general view. A key limitation is that respondents were only specifically asked if they held a saddle-fitting qualification, and what their professional memberships were. Hence, it was not possible to differentiate between individuals qualified in saddle fitting only, bridle and bit fitting only, and those with qualifications in both. Due to much smaller numbers of qualified bridle and bit fitters, they were grouped with saddle fitters for the purpose of statistical analysis.

This study has demonstrated that professionals within an MDT consider aspects of bridle and bit fit to be part of their role, but to a lesser extent than saddle fit [40]. There is overlap between professions regarding the key areas for improvement in bridle fit. For improvement in equine welfare, there is a need for quantitative evidence as to the prevalence of poor bridle/bit fit and suitability in UK ridden horses. Given the less transient nature of bridle and bit fit in comparison to the saddle, there seems to be scope to harness the combined efforts of the MDT and horse owners to raise standards in bridle and bit fit. Members of the MDT could be encouraged to increase their level of vigilance regarding bridle and bit fit and to know how to signpost horse owners should they wish to use professional bridle- and bit-fitting services. Whilst not advocating professionals cross professional boundaries, there is value in continuing professional development in bridle and bit fitting for saddle fitters, therapists and coaches alike so they are better equipped to support horse owners and, ultimately, better equipped to support the horse.

## 5. Conclusions

This study investigated how bridle and bit fit is assessed, managed, and acted upon by equestrian professionals. Despite differences in roles and responsibilities, the professionals identified similar key issues in bridle fit. This study suggests that various members of the equestrian MDT carry out some form of assessment of the bridle and bit as part of their professional role. The findings can be used to support equestrian professional practice, MDT effectiveness and, ultimately, improve bridle and bit fit for horses.

## Data Availability

Anonymized data are available on request.

## Supplementary Materials

The following supporting information can be downloaded at: https://www.mdpi.com/article/10.3390/ani14223188/s1, Supplementary File S1: Full survey provided.

## References

[1] Hartley Edwards E. (1990). Bitting in Theory and Practice.

[2] Bystrom A., Stalfelt A., Egenvall A., von Peinen K., Morgan K., Roepstorff L. (2010). Influence of girth strap placement and panel flocking material on the saddle pressure pattern during riding of horses. Equine Vet. J..

[3] Clayton H.M., O’Connor K., Kaiser L. (2014). Force and pressure distribution beneath a conventional dressage saddle and a treeless dressage saddle with panels. Vet. J..

[4] Fruehwirth B., Peham C., Scheidl M., Schobesberger H. (2004). Evaluation of pressure distribution under an English saddle at walk, trot and canter. Equine Vet. J..

[5] Latif S., von Peinen K., Wiestner T., Bitschnau C., Renk B., Weishaupt M. (2010). Saddle pressure patterns of three different training saddles (normal tree, flexible tree, treeless) in Thoroughbred racehorses at trot and gallop. Equine Vet. J..

[6] MacKechnie-Guire R., MacKechnie-Guire E., Fairfax V., Fisher D., Fisher M., Pfau T. (2019). The Effect of Tree Width on Thoracolumbar and Limb Kinematics, Saddle Pressure Distribution, and Thoracolumbar Dimensions in Sports Horses in Trot and Canter. Animals.

[7] Murray R., Guire R., Fisher M., Fairfax V. (2017). Reducing Peak Pressures Under the Saddle Panel at the Level of the 10th to 13th Thoracic Vertebrae May Be Associated With Improved Gait Features, Even When Saddles Are Fitted to Published Guidelines. J. Equine Vet. Sci..

[8] Murray R., Mackechnie-Guire R., Fisher M., Fairfax V. (2018). Reducing peak pressures under the saddle at thoracic vertebrae 10–13 is associated with alteration in jump kinematics. Comp. Exerc. Physiol..

[9] Nyikos S., Werner D., Müller J., Buess C., Keel R., Kalpen A., Vontobel H., von Plocki K., Auer J., von Rechenberg B. (2005). Measurements of saddle pressure in conjunction with back problems in horses. Pferdeheilkunde.

[10] Meschan E., Peham C., Schobesberger H., Licka T. (2007). The influence of the width of the saddle tree on the forces and the pressure distribution under the saddle. Vet. J..

[11] Clayton H.M. (2013). Science in brief: Interactions between the rider, the saddle and the horse. Equine Vet. J..

[12] Clayton H.M., Dyson S., Harris P., van Weeren R., Bondi A. (2019). Science-in-brief: Horse, rider, saddlery interactions: Welfare and performance. Equine Vet. J..

[13] Dyson S., Carson S., Fisher M. (2015). Saddle fitting, recognising an ill-fitting saddle and the consequences of an ill-fitting saddle to horse and rider. Equine Vet. Educ..

[14] Harman J. (1999). Tack and Sadde Fit. Vet. Clin. N. Am. Equine Pract..

[15] Harman J. (2004). The Horses’ Pain-Free Back and Saddle-Fit Book: Ensure Soundness and Comfort with Back Analysis and Correct Use of Saddles and Pads.

[16] Gertz E., Gebara K., Elbrønd V., Harrison A. (2020). The Effects of the Quantum and Finesse Bridles on Equine M. Brachiocephalicus and M. Splenius Function at Three Different Speeds. Open J. Vet. Med..

[17] Murray R., Guire R., Fisher M., Fairfax V. (2015). A Bridle Designed to Avoid Peak Pressure Locations Under the Headpiece and Noseband Is Associated with More Uniform Pressure and Increased Carpal and Tarsal Flexion, Compared with the Horse’s Usual Bridle. J. Equine Vet. Sci..

[18] Robinson N., Bye T. (2021). Noseband and poll pressures underneath bitted and bitless bridles and the effects on equine locomotion. J. Vet. Behav..

[19] Casey V., McGreevy P., O’Muiris E., Doherty O. (2013). A preliminary report on estimating the pressures exerted by a crank noseband in the horse. J. Vet. Behav..

[20] McGreevy P., Warren-Smith A., Guisard Y. (2012). The effect of double bridles and jaw-clamping crank nosebands on temperature of eyes and facial skin of horses. J. Vet. Behav..

[21] McGreevy P., Doherty O., Channon W., Kyrklund K., Webster J. (2017). The use of nosebands in equitation and the merits of an international equestrian welfare and safety committee: A commentary. Vet. J..

[22] Uldahl M., Clayton H.M. (2018). Lesions associated with the use of bits, nosebands, spurs and whips in Danish competition horses. Equine Vet. J..

[23] Visser E., Kuypers M., Stam J., Riedstra B. (2019). Practice of Noseband Use and Intentions Towards Behavioural Change in Dutch Equestrians. Animals.

[24] Merkies K., Copelin C., Small N., Young J. (2022). Noseband Fit: Measurements and Perceptions of Canadian Equestrians. Animals.

[25] Clayton H.M., Williams J.M. (2022). Know your noseband: An exploration of factors that influence riders’ choice of noseband. J. Vet. Behav..

[26] Clayton H.M. (1985). A fluoroscopic study of the position and action of different bits in the horse’s mouth. J. Equine Vet. Sci..

[27] Manfredi J., Clayton H.M., Rosenstein D. (2005). Radiographic study of bit position within the horse’s oral cavity. Equine Comp. Exerc. Physiol..

[28] Manfredi J., Rosenstein D., Lanovaz J., Nauwelaerts S., Clayton H.M. (2010). Fluoroscopic study of oral behaviours in response to the presence of a bit and the effects of rein tension. Comp. Exerc. Physiol..

[29] Tuomola K., Maki-Kihnia N., Valros A., Mykkanen A., Kujala-Wirth M. (2021). Bit-Related Lesions in Event Horses After a Cross-Country Test. Front. Vet. Sci..

[30] Tuomola K., Maki-Kihnia N., Valros A., Mykkanen A., Kujala-Wirth M. (2021). Risk factors for bit-related lesions in Finnish trotting horses. Equine Vet. J..

[31] World Horse Welfare Bridles: How to Choose and Fit Them Correctly. https://www.worldhorsewelfare.org/advice/bridles-how-to-choose-and-fit-them-correctly?srsltid=AfmBOor_mQR0wcbR-_teH8yjv34KAddULhQpNqdUZVG569hIs2-klsHN.

[32] MacKechnie-Guire R., Williams J.M., Fisher D., Nankervis K. (2024). The Role of Equestrian Professionals in Saddle Fit for Horses and Riders in the United Kingdom. Animals.

[33] Society of Master Saddlers Website. https://www.mastersaddlers.co.uk/members/.

[34] Taberna M., Gil Moncayo F., Jane-Salas E., Antonio M., Arribas L., Vilajosana E. (2020). The Multidisciplinary Team (MDT) Approach and Quality of Care. Front. Oncol..

[35] Nankervis K., MacKechnie-Guire R., Maddock C., Pyatt A. (2024). Experiences of Interdisciplinary Working from the Perspective of the Society of Master Saddlers Qualified Saddle Fitters. Animals.

[36] Lynden J., Ogden J., Hollands T. (2018). Contracting for care—The construction of the farrier role in supporting horse owners to prevent laminitis. Equine Vet. J..

[37] Hesse K., Verheyen K. (2010). Associations between physiotherapy findings and subsequent diagnosis of pelvic or hindlimb fracture in racing Thoroughbreds. Equine Vet. J..

[38] Greve L., Dyson S. (2014). The interrelationship of lameness, saddle slip and back shape in the general sports horse population. Equine Vet. J..

[39] Dittmann M., Arpagaus S., Hungerbuhler V., Weishaupt M., Latif S. (2021). “Feel the Force”-Prevalence of Subjectively Assessed Saddle Fit Problems in Swiss Riding Horses and Their Association With Saddle Pressure Measurements and Back Pain. J. Equine Vet. Sci..

[40] Greve L., Dyson S. (2015). A longitudinal study of back dimension changes over 1 year in sports horses. Vet. J..

[41] Equine Business Association Equine Industry Statistics. https://equinebusinessassociation.com/equine-industry-statistics/#:~:text=With%20over%2018%2C000%20equine%20businesses,front%20of%20this%20large%20audience.

[42] FEI Judging Manual. https://inside.fei.org/sites/default/files/FEI%20Dressage%20Judging%20Manual%20-%20Effective%201%20January%202024_0.pdf.

[43] Warren-Smith A.K., McGreevy P.D. (2007). The use of blended positive and negative reinforcement in shaping the halt response of horses (*Equus caballus*). Anim.Welf..

[44] Scrutchfield W., Baker G.J., Easley J. (1999). Equine dental instrumentation. Equine Dentistry.

[45] Dyson S. (2022). The ridden horse pain ethogram. Equine Vet. Educ..

[46] Dyson S., Bondi A., Routh J., Pollard D., Preston T., McConnell C. (2021). An investigation of behaviour during tacking-up and mounting in ridden sports and leisure horses. Equine Vet. Educ..

[47] Commission EEaW A Good Life for Horses—A Vision for the Future Involvement of Horses in Sport. Final Report—November 2023..

[48] Commission EEaW (2023). Equine Ethics and Wellbeing Commission—24 Draft Recommendations. https://equinewellbeing.fei.org/assets/documents/EEWB%2024%20Draft%20Recommendations.pdf.

[49] The Equine Fitters Council. https://www.equinefittersdirectory.org/what-we-do/.

